# *Streptococcus gallolyticus* endocarditis on a prosthetic tricuspid valve: a case report and review of the literature

**DOI:** 10.1186/s13256-021-03125-5

**Published:** 2021-10-27

**Authors:** Raz Shapira, Tamir Weiss, Elad Goldberg, Eytan Cohen, Ilan Krause, Ram Sharony, Idan Goldberg

**Affiliations:** 1 Sackler Faculty of Medicine, Tel Aviv University, Tel Aviv, Israel; 2 The Department of Medicine F - Recanati, Rabin Medical Center (Beilinson Campus), Petah Tikva, Israel; 3 The Department of Cardiothoracic Surgery, Rabin Medical Center (Beilinson Campus), Petah Tikva, Israel; 4 Institute of Hematology, Davidoff Cancer Center, Rabin Medical Center, Petah Tikva, Israel

**Keywords:** *Streptococcus bovis*, *Streptococcus gallolyticus*, Infective endocarditis, Prosthetic tricuspid valve, Case report

## Abstract

**Background:**

*Streptococcus gallolyticus* subspecies *gallolyticus* is a known pathogen that causes infective endocarditis, and most cases involve the left heart valves. We present the first reported case of prosthetic tricuspid valve endocarditis caused by this microorganism. Relevant literature is reviewed.

**Case presentation:**

A 67-year-old Jewish female with a history of a prosthetic tricuspid valve replacement was admitted to the emergency department because of nonspecific complaints including effort dyspnea, fatigue, and a single episode of transient visual loss and fever. No significant physical findings were observed. Laboratory examinations revealed microangiopathic hemolytic anemia and a few nonspecific abnormalities. Transesophageal echocardiogram demonstrated a vegetation attached to the prosthetic tricuspid valve. The involved tricuspid valve was replaced by a new tissue valve, and *Streptococcus gallolyticus* subspecies *gallolyticus* was grown from its culture. Prolonged antibiotic treatment was initiated.

**Conclusions:**

Based on this report and the reviewed literature, *Streptococcus gallolyticus* should be considered as a rare but potential causative microorganism in prosthetic right-sided valves endocarditis. The patient’s atypical presentation emphasizes the need for a high index of suspicion for the diagnosis of infective endocarditis.

## Background

Infective endocarditis (IE) is defined as an infection of the endocardial surface of the heart, and most commonly involves the heart valves. Less commonly, ventricular septal defect, mural endocardium, or intracardiac devices may also be involved. Right-sided endocarditis accounts for 5–10% of IE cases, and is strongly associated with intravenous drug abusers (IVDA) [1]. Most cases involve the tricuspid valve, and *Staphylococcus aureus* (*S. aureus)* is the most common pathogen [1, 2]. Unlike left-sided IE, isolated right-sided IE is not associated with peripheral embolic and vascular manifestations, unless there is an intracardiac shunt. Pulmonary findings, such as lung abscesses, may be present [3].

*Streptococcus gallolyticus* subspecies *gallolyticus* (*S. gallolyticus*), formerly *Streptococcus bovis* biotype 1, is part of the *Streptococcus bovis *(*S. bovis*) complex, which is responsible for 2–10% of IE cases [2]. IE associated with the *S. bovis* complex most commonly involves the aortic valve, the mitral valve, or both. Involvement of the tricuspid valve or pacemaker electrode is very rare [2, 7–23]. In this report, we use the name *S. bovis* to discuss the results of studies that did not relate to the specific subspecies of that complex.

We present here an atypical case of *S. gallolyticus* infection of a prosthetic tricuspid valve and a pacemaker electrode in a non-IVDA woman.

## Case presentation

A 67-year-old Jewish female was admitted to our hospital because of worsening effort dyspnea and cough, extreme fatigue, and functional decline. Her complaints began 2 months prior to admission, following a single episode of fever (38 °C), transient bilateral loss of sight, and vomiting. Her medical history includes a bioprosthetic tricuspid valve implantation 4 years prior to the current hospitalization due to tricuspid stenosis, followed by a pacemaker implantation due to periprocedural complete heart block. Her regular medical therapy includes allopurinol (100 mg per day), bisoprolol (2.5 mg per day), apixaban (2.5 mg twice a day), and vitamin D3 (1000 international units per day). The patient had not undergone recent medical procedures (including dental care) and had no history of intravenous drug use.

**Fig. 1:**
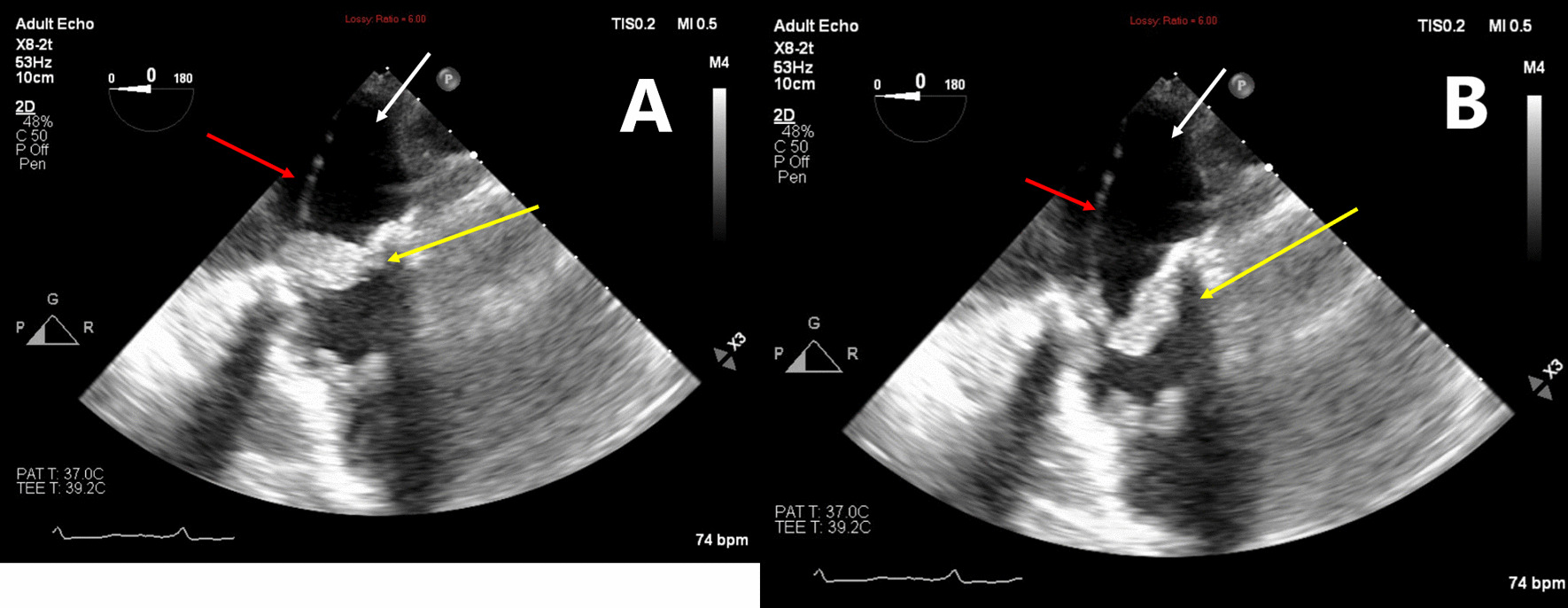
Transesophageal echocardiogram images during systole (**a**) and diastole (**b**). Large vegetation is attached to the pacemaker electrode (red arrow) and the prosthetic tricuspid valve (yellow arrow), resulting in severe tricuspid stenosis. The white arrow points to the right atrium

At the time of her admission, the patient was alert and afebrile, and presented normal vital signs. Physical examination demonstrated no significant findings, without peripheral stigmata of endocarditis, heart murmurs, or neurological deficits. Initial laboratory tests revealed mild hemolytic anemia [decrease in hemoglobin from a recent level of 14.9 g/dL to 12.2 g/dL, increased lactate dehydrogenase (LDH) levels up to 1080 U/L, and mild indirect hyperbilirubinemia], in addition to the presence of schistocytes in the peripheral blood smear. Additional abnormal laboratory results included increased levels of C-reactive protein (CRP) and an elevation of cholestatic liver enzymes. Urinalysis demonstrated hematuria and mild proteinuria, without clinical or laboratory findings of acute kidney injury. A mildly elevated level of rheumatoid factor was noted. Chest X-ray and fundoscopy revealed no pathological findings. Transthoracic echocardiogram demonstrated a 1.1 cm vegetation on the tricuspid valve. Transesophageal echocardiogram demonstrated severe tricuspid stenosis as a complication of the attached vegetation, as well as an additional 1.1 cm vegetation on the pacemaker electrode (Fig. 1). Fluorodeoxyglucose positron-emission tomography–computed tomography (FDG PET-CT) demonstrated pathological rectal uptake, suggestive of a neoplastic process. No septic pulmonary emboli were observed. During the first 2 days of hospitalization, a total of five blood culture sets were positive for *S. gallolyticus*, and susceptible for beta-lactam antibiotics as well as for clindamycin, erythromycin, and vancomycin. The minimum inhibitory concentration (MIC) for penicillin and ampicillin was 0.094 µg/mL. Intravenous therapy with ceftriaxone was initiated, according to the antibiotic sensitivity profile of the pathogen.

**Fig. 2:**
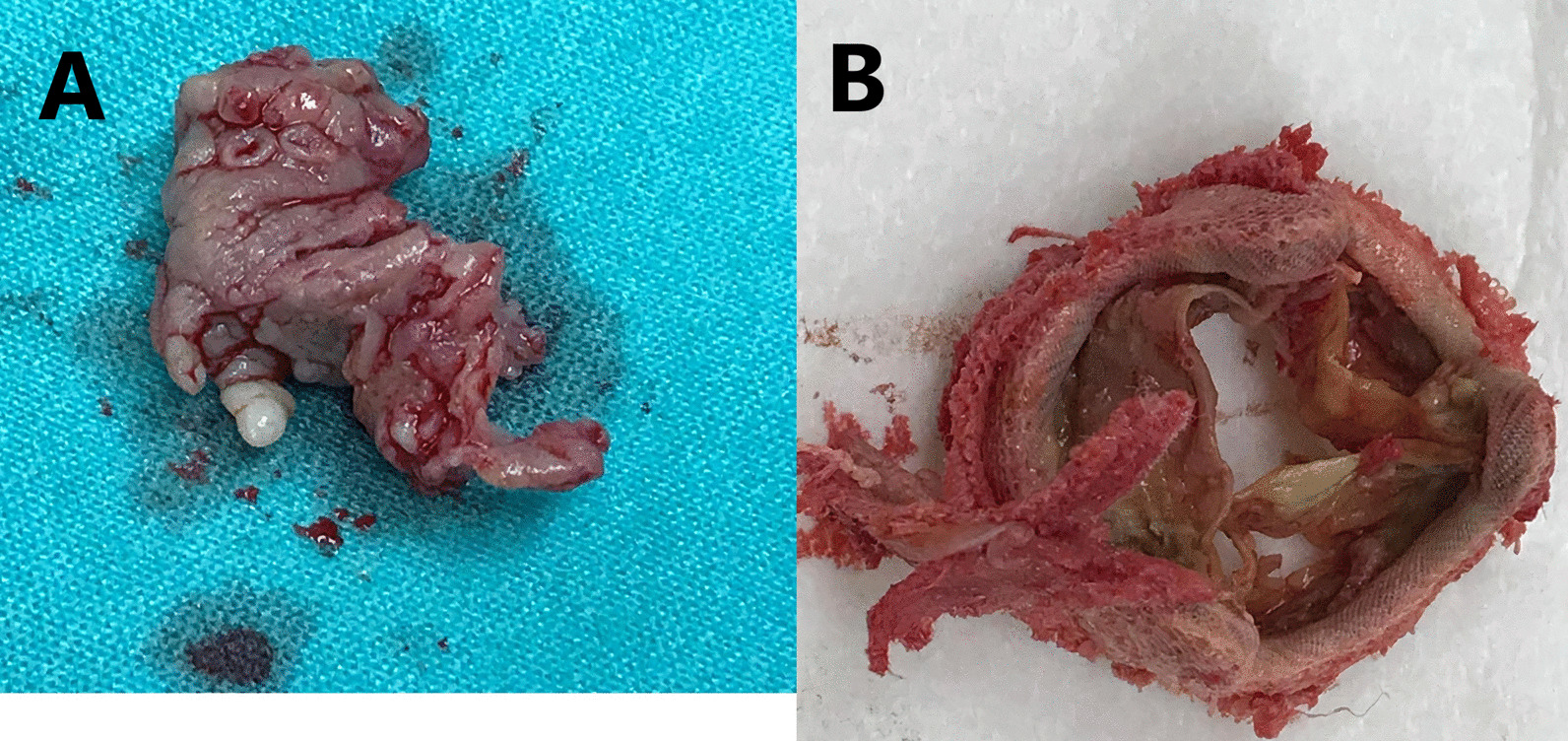
Intraoperative images. **a** Large vegetation; **b** infected prosthetic tricuspid valve

On day 11 after admission, open-heart surgery was performed. The involved tricuspid valve was replaced (Fig. 2) by a tissue valve (Mosaic 27 by Medtronic). The infected pacemaker electrodes were removed and replaced by temporary, and later permanent, epicardial electrodes. The intraoperative and postoperative course was uneventful. Positive growth of *S. gallolyticus* was obtained from both the removed valve and electrodes. The patient was treated with 2 g of ceftriaxone for an additional 6 weeks. After a rehabilitation process in our hospital, the patient was discharged for further ambulatory follow-up, including endoscopic evaluation of the rectal lesion mentioned above. To the best of our knowledge, the patient refused to undergo this evaluation because of personal reasons. Three years after her discharge, the patient is clinically stable, with no clinical or laboratory findings that raise suspicion of rectal malignancy.

## Discussion and conclusions

To the best of our knowledge, this is the first reported case of *S. bovis* IE on a prosthetic tricuspid valve. Table 1 summarizes an extensive literature review including 16 studies and a total of 500 cases of *S. bovis* IE. In these studies, *S. bovis* was responsible for about 10% of the total IE cases. The vast majority of cases involved native valves (87%). A native aortic valve was the most prevalent site of infection (28%), followed by a coinfection of native aortic and mitral valves (18%). An isolated infection of a native mitral valve was reported in 16% of the cases and a native tricuspid valve infection in only 2%. Prosthetic tricuspid valve involvement has not been reported in any of the cases, or in any other case report. Furthermore, infection of pacemaker electrodes was also extremely rare, with only two reports in the reviewed studies (0.4%) and a few case reports.

**Table 1:** Anatomical characteristics of *S. bovis* IE cases: review of the literature [7–23]

References	*S. bovis* cases (% from total cases)	Native valves						Prosthetic valves			PM
		Ao	Mt	Ao and Mt	Tr	Others	Total	Left side	Right side	Total	
Sidda *et al.* (2018) [18]	8^a^ (N/D)	0	5 (62.5%)	0	0	0	5 (62.5%)	3 (37.5%)	**0**	3 (37.5%)	2 (25%)^b^
Mello *et al.* (2015) [15]	9 (4.5%)	6 (67%)	0	2 (22%)	0	1 (11%)	9 (100%)	0	**0**	0	0
García-País *et al.* (2015) [22]	104^c^ (N/D)	30 (29%)	23 (22%)	28 (27%)	1 (1%)	N/D	82 (79%)	22 (21%)	**0**	22 (21%)	0^d^
Mohee *et al.* (2014) [19]	36 (9%)	N/D^e^	N/D^e^	N/D^e^	0	0	27 (75%)^e^	9 (25%)	**0**	9 (25%)	0
Fitzmaurice *et al.* (2013) [14]	7 (N/D)	1 (14%)	1 (14%)	1 (14%)	0	1 (14%)	4 (57%)	3 (43%)	**0**	3 (43%)	0
CorredoIra *et al.* (2008) [7]	55 (24%)	N/D^f^	N/D^f^	N/D^f^	0	0	47 (85%)^f^	8 (14.5%)	**0**	8 (14.5%)	0
Tripodi *et al.* (2004 and 2005) [16, 23]	52 (in 2004, 15%; in 2005, N/D)	17 (33%)	7 (13%)	19 (36.5%)	3 (6%)	1 (2%)	47 (90%)	5 (10%)	**0**	5 (10%)	0
González juanatey *et al.* (2003) [17]	20 (17%)	8 (40%)	1 (5%)	11 (55%)	0	0	20 (100%)	0	**0**	0	0
Massaroni *et al.* (2003) [11]	47 (4.5%)	20 (42.5%)	16 (34%)	5 (11%)	1 (2%)	0	42 (89%)	5 (11%)	**0**	5 (11%)	0
Herrero *et al.* (2002) [13]	13 (N/D)	5 (42%)	2 (8%)	3 (25%)	0	2 (17%)	12 (92%)	1 (8%)	**0**	1 (8%)	0
Duval *et al.* (2001) [8]	20 (N/D)	8 (40%)	9 (45%)	2 (10%)	1 (5%)	0	20 (100%)	0	**0**	0	0
Pergola *et al.* (2001) [12]	40 (19%)	15 (37.5%)	7 (17.5%)	N/D	2 (5%)	11 (27.5)	35 (87.5%)	N/D	N/D	5 (12.5%)	0
Carfagna *et al.* (1998) [10]	14 (N/D)	N/D^g^	N/D^g^	0	0	0	13 (93%)^g^	1 (7%)	**0**	1 (7%)	0
Ballet *et al.* (1995) [9]	53 (11%)	26 (50%)	6 (11%)	18 (34%)	1 (2%)	2 (4%)	53 (100%)	0	**0**	0	0
Beeching *et al.* (1985) [21]	10 (N/D)	3 (30%)	3 (30%)	0	1 (10%)	1 (10%)	8 (80%)	2 (20%)	**0**	2 (20%)	0

*Ao* aortic valve, *Mt* mitral valve, *Tr* tricuspid valve, *PM* pacemaker, *N/D* no data

^a^This study describes 12 patients with diagnosed endocarditis, but anatomical information is reported for 8 patients only. The authors confirmed that none of the other cases had involved a tricuspid valve infection

^b^Both cases include coinfection of a pacemaker lead and the mitral valve

^c^This study describes 89 native valve and 23 prosthetic valve IE cases, but anatomical information is reported for only 82 and 22 cases, respectively. No case of prosthetic tricuspid valve infection is reported in the study

^d^This group published another study with very similar findings that is not included in Table 1 [20]. That study reported a single case of a pacemaker IE among 109 patients with *S. bovis* IE. There were no cases of prosthetic tricuspid valve IE

^e^All patients in this study had left valves infection (18 in the aortic valve and 19 in the mitral valve). Twenty-seven of these cases involved native valves, and nine involved prosthetic valves

^f^All patients in this study had left valves infection (22 in the aortic valve, 10 in the mitral valve, and 23 in both valves). Forty-seven of these cases involved native valves, and eight involved prosthetic valves

^g^All patients in this study had left valves infection (eight in the aortic valve and six in the mitral valve). Thirteen of these cases involved native valves, and one involved prosthetic valve

*Streptococcus gallolyticus* infections are also associated with colonic neoplasms. A recent study from a multicenter registry found colorectal tumors in 69% of *S. bovis* IE patients, with a clear predominance of benign lesions (78%) [24]. Among the four subspecies of the *S. bovis* complex*, S. gallolyticus* has the strongest association with colonic neoplasm, and this association is higher for IE compared with other sites of *S. gallolyticus* infection [25]. Therefore, gastrointestinal endoscopy may be advisable as routine screening for occult gastrointestinal lesions in patients with *S. bovis* bacteremia.

A history of prosthetic heart valve implantation constitutes a significant risk factor for IE, with the greatest risk during the first 6–12 months after the valve replacement. Prosthetic valve endocarditis (PVE) accounts for 7–25% of IE cases in developed countries, with a similar risk for bioprosthetic and mechanical valves at 5 years after the valve replacement [3]. Antecedent native valve IE, implantation of multiple valves, male sex, and older age were reported as risk factors for PVE [4–6].

As mentioned before, *S. aureus* is the most common pathogen causing tricuspid IE, a disease that primarily affects IVDA [1]. Besides IVDA, right-sided IE can also occur in patients with intracardiac devices (such as a pacemaker electrode), as in this case. In 2016, the European Society of Cardiology reported a threefold increase in the incidence of pacemaker IE among non-IVDA over the past 26 years. According to the study, pacemaker IE represented 6.1% of all IE cases, and most of the affected patients were males (80%), without a history of underlying structural heart disease (92%) or previous IE. The common pathogens were staphylococci and enterococci (84% and 12%, respectively). No cases of *S. bovis* pacemaker IE were observed [26]. In another large multicenter prospective cohort study, *S. bovis* was found in only 3% of patients with IE involving intracardiac electronic devices (including pacemakers and implantable cardioverter defibrillators) [2].

IE is a complex infectious disease characterized by a highly variable clinical presentation, heterogeneous patient populations, and various causative microorganisms. The patient presented here was admitted to the emergency room because of nonspecific complaints, and her physical examination demonstrated no significant findings. Initial laboratory tests revealed mild hemolytic anemia and presence of schistocytes in peripheral blood smear. In the absence of fever, heart murmurs, or peripheral IE manifestations, the existence of new mechanical hemolysis in a patient with a biological prosthetic valve was the major clinical hint for IE and prompted the need to obtain blood cultures in the emergency room. This case demonstrates that IE should be suspected and ruled out in cases of new or worsening hemolysis in patients with intracardiac risk factors for IE. Based on this report and the reviewed literature, *S. bovis* should be considered as a rare but potential causative organism in cases of right-sided IE.

## Data Availability

Data sharing is not applicable to this article as no datasets were generated or analyzed during the current study.

## Abbreviations

IE: Infective endocarditis
IVDA: Intravenous drug abuser
*S. aureus*: *Staphylococcus aureus*
*S. gallolyticus*: *Streptococcus gallolyticus* subspecies *gallolyticus*
*S. bovis*: *Streptococcus bovis*
LDH: Lactate dehydrogenase
CRP: C-reactive protein
FDG PET-CT: Fluorodeoxyglucose positron-emission tomography–computed tomography
